# A Validated ^1^H NMR Method for the Quantitation of 5-Hydroxymethyl-2(5H)-Furanone, a Valuable Chemical Intermediate, In a Dichloromethane Extract of *Helleborus lividus* subsp: C*orsicus* Leaves from Corsica

**DOI:** 10.1155/2022/9580338

**Published:** 2022-08-24

**Authors:** Thomas Maroselli, Mathieu Paoli, Ange Bighelli

**Affiliations:** Université de Corse-CNRS, Équipe Chimie et Biomasse, UMR 6134 SPE, Ajaccio, France

## Abstract

An experimental procedure using ^1^H NMR was developed and validated to quantify 5-hydroxymethyl-2(5H)-furanone, a valuable chemical synthon ((S)-enantiomer), in a dichloromethane extract of *Helleborus lividus* subsp. *corsicus* leaves. This method, using vanillin as the internal standard, exhibited a perfect linearity of measurements (*R*^2^ = 1) associated with very good accuracy (relative errors comprised between −1.62% and 4.25%) and precision (reproducibility 30.51 mg ± 0.4%). The limit of detection and the limit of quantitation have been measured at 0.14 mg and 0.59 mg, respectively. The experiment time is very short since a single analysis is at the minute level. 5-Hydroxymethyl-2(5H)-furanone accounted for nearly 85% in the dichloromethane extract of *H. lividus* subsp. *corsicus* leaves (1.7% of the mass of fresh leaves). This plant represents an important and natural source of (S)-5-hydroxymethyl-2(5H)-furanone (main enantiomer; determined using a GC chiral analysis).

## 1. Introduction

(S)-5-Hydroxymethyl-2(5H)-furanone is a versatile chemical intermediate used for the syntheses of a wide range of natural and/or bioactive compounds such as norrisolide, trans-burseran, or steganacin [1–3]. Its reactivity, applications, and its own syntheses have been extensively investigated for forty years and the reported results have been recently reviewed by Flourat et al. [4]. Synthetic pathways leading to (S)-5-hydroxymethyl-2(5H)-furanone have significantly evolved since the end of the 70's and could be now achieved by applying green chemistry principles [4]. This molecule, rarely found as a natural compound, was reported for the first time, at very low content, in a *Clematis hirsuta* (Ranunculaceae) butanol extract after purification [5].

In the course of our ongoing work concerning the valorization of medicinal and aromatic plants growing wild in Corsica, we got interested by *Helleborus lividus* subsp. *corsicus* (Ranunculaceae). This endemic plant, listed as toxic by the Conservatoire Botanique de Corse, was not yet investigated for its phytochemicals. *H. lividus* subsp. *corsicus* usually grows near rivers, on grassy, or rocky slopes up to 1500 m. Its thick stems, 70 cm to 1 m high, carry alternate leaves divided in three lanceolate leaflets with fine-toothed margins. The upper limb side is dark green, whereas, the lower side is clearer. When growing, this perennial plant produces a massive and persistent foliage clump. At maturity, each stem produces a tall and ramified inflorescence. The flower perianth is flat and circular, and is composed of five yellow to green-apple tepals [6].

In the present paper, we report on the identification by ^13^C NMR spectroscopy, in a dichloromethane extract of *H. lividus* subsp. *corsicus* leaves, of 5-hydroxymethyl-2(5H)-furanone as the main component. Therefore, with respect to the many synthetic uses of this compound, we got interested to appreciate its content in *H. lividus* subsp. *corsicus*. The quantitation was achieved by ^1^H NMR, after validation of the method. This technique, that allows a short time of occupancy of the spectrometer, is not yet investigated for this compound.

## 2. Results and Discussion

**Figure 1:**
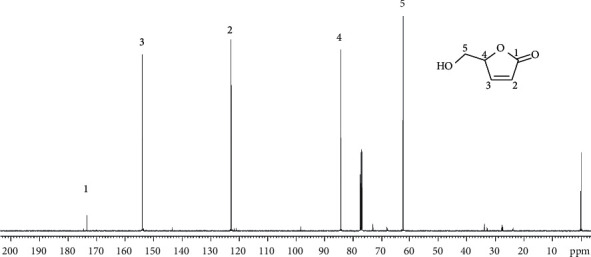
^13^C NMR spectrum of the *Helleborus lividus* subsp. *corsicus* dichloromethane leaf extract.

Leaves of *H. lividus* subsp. *corsicus* harvested in the Vizzavona forest (Corsica) have been successively extracted with hexane, in order to eliminate long chain fatty acids, and then with dichloromethane. The ^13^ C NMR spectrum of the dichloromethane extract displayed a series of five signals of very strong intensities corresponding by far to the major component (Figure 1). They were differentiated by DEPT experiments as a carbonyl (173.52 ppm), two olefinic methines (153.97 ppm and 122.86 ppm), one oxygenated methine (84.31 ppm), and one oxymethylene (62.18 ppm). The NMR data suggested the molecular formula C_5_H_6_O_3_. According to the three degrees of insaturation, this compound contains one ring. In order to establish its structure, 2D NMR experiments (HSQC, HMBC, and COSY) were conducted on the extract. The COSY spectrum showed correlations between the O-CH proton at 5.17 ppm, both the oxymethylene protons at 4.01 ppm and 3.80 ppm, and the olefinic proton at 7.50 ppm; the latter correlated with the second olefinic proton at 6.16 ppm. These data evidenced a five-membered *α*, *β*-unsaturated lactone with hydroxymethyl connected to the oxygenated methine: 5-hydroxymethyl-2(5H)-furanone (Table 1). This structure is in agreement with HMBC correlations. Identification of 5-hydroxymethyl-2(5H)-furanone was confirmed by comparison of its NMR data with those reported in the literature [7–10]. Moreover, we recorded the ^1^H and ^13^C spectrum of pure (commercial) (S)-5-hydroxymethyl-2(5H)-furanone and the chemical shift values fitted perfectly with those of the main component of the dichloromethane extract. In order to determine the enantiomeric ratio of this chiral compound, the *H. lividus* subsp. *corsicus* dichloromethane leaf extract was submitted to an enantioselective capillary GC analysis. The studied sample contained 96.90% of (S)-enantiomer and 3.10% of (R)-enantiomer.

In the ^13^C NMR spectrum of this extract, various signals with weak intensities remained unassigned. Comparison of the chemical shift values with literature data allowed the identification of two minor components: protoanemonin (*δ*^13^C NMR: 170.11 ppm (C=O), 154.92 ppm (C), 143.49 ppm (CH), 121.72 ppm (CH), and 98.31 ppm (CH_2_)) and anemonin (*δ*^13^C NMR: 170.11 ppm (C=O), 153.57 ppm (CH), 121.06 ppm (CH), 90.41 ppm (C), and 23.82 ppm (CH_2_)). The NMR data of these two compounds fit with those reported by Southwell and Tucker [11], and Kern and Cardellina [12]. Protoanemonin, an irritant lactone, and its dimerization product, anemonin, are characteristic constituents of Ranunculaceae species such as *H. Niger* [13] or *H. foetidus* [14].

In our laboratories, quantitation of compounds such as lignans in *Cedrus atlantica* resins [15] as well as ursolic and oleanolic acids in a dichloromethane extract from *Ilex aquifolium* [16] has been achieved using ^1^H NMR, with a time of occupancy of the spectrometer at the minute level. Therefore, quantitation of 5-hydroxymethyl-2(5H)-furanone, the main compound of the *H. lividus* subsp. *corsicus* dichloromethane leaf extract, has been undertaken with this technique.

Quantitative determination of a component in a mixture can be performed by internal calibration that requires the comparison of the area(s) of one or various signal(s) of that compound with those of an internal standard. NMR spectroscopy allows the quantitation of a component by integration of one of its signals since the area value is proportional to the number of nuclei. Internal calibration requires a homogenous answer of selected nuclei of the compound and an internal reference. Two parameters are responsible of the answer (uniform or nonuniform) of nuclei: (i) the longitudinal relaxation time (*T*_1_) of nuclei and (ii) the differences in the nuclear overhauser enhancement (NOE) between selected nuclei of the component and those of reference [16].

The signals of the two ethylenic protons of 5-hydroxymethyl-2(5H)-furanone (dd, 6.16 ppm and dd, 7.50 ppm) are perfectly resolved and do not overlap with those of the minor components of the *H. lividus* subsp. *corsicus* leaf dichloromethane extract such as anemonin and protoanemonin. Therefore, these signals could be separately used to quantify 5-hydroxymethyl-2(5H)-furanone. Deuterated chloroform (CDCl_3_) has been chosen as the solvent and vanillin as the internal reference. Indeed, signals of vanillin (particularly the singlet of the aldehydic proton at 9.77 ppm) do not overlap with those of 5-hydroxymethyl-2(5H)-furanone (and minor compounds of the extract).

*T*_1_ value of the two ethylenic protons of 5-hydroxymethyl-2(5H)-furanone has been measured by the inversion-recovery method in CDCl_3_ (*T*_1_ = 0.5 s for both protons) and compared to that of the aldehydic proton of vanillin in the same solvent (*T*_1_ = 0.9 s) [17]. The percentage of recovered signal S : N (%) was determined and plotted according to Becker et al. [18] as a function of the pulse angle *α* for the selected protons with *T*_1_ = 0.5 s and 0.9 s. The difference of the steady-state magnetization is extremely small for a flip angle of 30°, *i.e.,* ΔMz = 0.1.

Then, we got interested to know if the pulse sequence usually used for recording the ^1^H NMR spectra (flip angle *α* = 30°, *D*_1_ = 1.0 s, a total recycling time of 3.56 s; as shown in the experiment) was well suited for the quantitative determination of 5-hydroxymethyl-2(5H)-furanone in a leaves dichloromethane extract of *H. lividus* subsp*. corsicus*. We previously used this sequence to quantify oleanolic and ursolic acids in an *I. aquifolium* extract [16].

Accuracy, response linearity, and precision of this method have been validated by various experiments carried out using commercial 5-hydroxymethyl-2(5H)-furanone (purity: 98%), taking into account the relative areas of the aldehydic proton of vanillin and those of each ethylenic proton of 5-hydroxymethyl-2(5H)-furanone (contents calculated using formula).

(1) $$\begin{array}{c} m_{F}=\frac{\;A_{F}\times M_{F}\times m_{V}\times P_{V}}{A_{V}\times M_{V}\times P_{F}}, \end{array}$$

*m*_*F*_: calculated mass (mg) of 5-hydroxymethyl-2(5H)-furanone; *A*_*F*_ and *M*_*F*_: area of the selected ethylenic signal and molecular weight (114.10 g mol^−1^) of 5-hydroxymethyl-2(5H)-furanone; *m*_*V*_, *M*_*V*_, and *A*_*V*_: amount (mg), molecular weight (152.15 g·mol^−1^), and area of the signal of the aldehydic proton of vanillin (*A*_*v*_ fixed at 1.00 in all experiments); *P*_*V*_ and *P*_*F*_: purity of vanillin (99%) and 5-hydroxymethyl-2(5H)-furanone (98%), respectively.

Accuracy of the procedure was determined by comparing different weighted amounts of pure 5-hydroxymethyl-2(5H)-furanone (2.03 mg-30.51 mg) with those measured by ^1^H NMR (Table 2). All the experiments have been recorded in the presence of 12.60 mg of vanillin in 0.5 mL of CDCl_3_. The relative errors, comprised between −1.62% and 4.25%, demonstrated a good accuracy of measurements.

**Figure 2:**
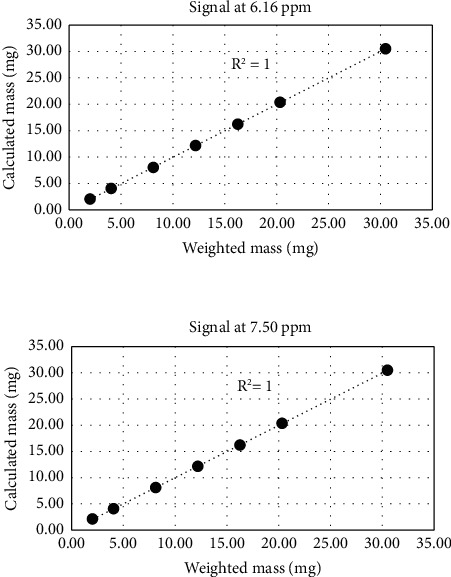
Response linearity of quantitation of 5-hydroxymethyl-2(5H)-furanone using ^1^H NMR. Aldehydic proton of vanillin. Ethylenic protons of 5-hydroxymethyl-2(5H)-furanone.

Then, the calibration line (linearity) for the quantitation of 5-hydroxymethyl-2(5H)-furanone has been plotted by expressing the ratio of the calculated mass of this compound as a function of the weighted one. We observed a perfect linearity of the measurements with the linear determination factor *R*^2^ = 1 (Figure 2).

Afterwards, five spectra of the same sample containing 5-hydroxymethyl-2(5H)-furanone (30.51 mg) and vanillin (12.60 mg) have been recorded with the same experimental conditions to check the precision of the procedure. The measured mass of 5-hydroxymethyl-2(5H)-furanone, calculated using formula (1), were practically constant (30.49–30.58 mg). The relative errors between the calculated mass and the weighted one varied between −0.07% and 0.23% (Table 3). The reproducibility (tube to tube variability) of 30.51 mg ± 0.4%, calculated with a confidence interval of 99% (Student's *t*-test), indicated a good precision of measurements.

Finally, the limit of detection (LOD) and the limit of quantification (LOQ) (as shown in the experiment) have been measured at 0.14 mg and 0.59 mg, respectively.

**Figure 3:**
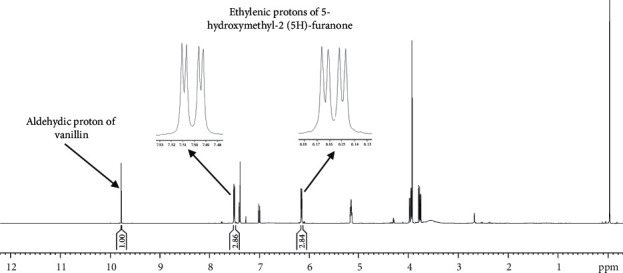
^1^H NMR spectrum of the *Helleborus lividus* subsp. *corsicus* dichloromethane leaf extract.

The quantitative procedure using ^1^H NMR is being validated taking into account separately the two ethylenic protons of 5-hydroxymethyl-2(5H)-furanone; this technique has been used to calculate its content in the *H. lividus* subsp. *corsicus* dichloromethane leaf extract (Table 4, Figure 3). That compound (enantiomeric ratio (S : R): 97 : 3, calculated using a GC chiral analysis) represents nearly 85% of the crude extract. Therefore, the investigated leaves contained approximately 1.7% of (S)-5-hydroxymethyl-2(5H)-furanone.

As a conclusion, a rapid experimental procedure, based on ^1^H NMR spectroscopic analysis (flip angle *α* = 30°, *D*_1_ = 1.0 s, total recycling time 3.56 s) was developed and allowed the quantitation of 5-hydroxymethyl-2(5H)-furanone, the major component of a dichloromethane leaf extract of *H. lividus* subsp. *Corsicus*. This plant may be considered as a natural source of (S)-5-hydroxymethyl-2(5H)-furanone (main enantiomer), a compound regularly used in the synthesis of natural and/or pharmaceutical products. This compound was purified up to 98% (quantitation by ^1^H NMR) from the dichloromethane extract, using an automated flash chromatrograph (as shown in the experiment). However, the chromatographic process could be improved in order to be used for pharmaceutical purposes.

## 3. Material and Methods

### 3.1. Plant Material and Solvent Extractions

Leaves from *H. lividus* subsp. *corsicus* (individual plant) have been harvested in January 2020, in the Vizzavona forest (center of Corsica: 42°06′43.6″N; 9°06′50.1″E). Leaves (143.4 g) have been crushed in liquid nitrogen and then successively extracted (soxhlet apparatus) with hexane (600 mL, 48 h) and dichloromethane (600 mL, 48 h). The solvent has been removed under reduced pressure yielding 843.2 mg of hexane extract (yield: 0.58%) and 2.888 g of dichloromethane extract (yield: 2.01%), respectively. Yields have been calculated from fresh material (w : w).

### 3.2. Dichloromethane Extract Fractionation

The dichloromethane extract from *H. lividus* subsp. *corsi*cus leaves (912.2 mg) was submitted to flash chromatography using an Interchim PuriFlash 4250 automated system (UV detection and evaporative light scattering detector; silica cartridge F023, 23 g, 50 *μ*m; 20 bar). Seven fractions were obtained with a mixture of solvents of increasing polarity (pentane/dichloromethane/ethyl acetate/methanol): F1 (100/0/0/0) = 3.4 mg; F2 (40/60/0/0) = 30.6 mg; F3 (30/70/0/0) = 237.0 mg; F4 (0/100/0/0) = 187.2 mg; F5 (0/90/10/0) = 235.0 mg; F6 (0/85/15/0) = 93.7 mg; F7 (0/0/50/50) = 59.5 mg. Fractions of chromatography were analyzed by ^1^H and ^13^C NMR. 5-Hydroxymethyl-2(5H)-furanone was purified in F3 (98%).

### 3.3. GC Chiral Analysis Conditions

The enantioselective capillary GC analysis was carried out using a Clarus 500 Perkin Elmer system equipped with a FID and a fused-silica capillary column (30 m, i.d. 0.25 mm, film thickness 0.25 *µ*m), *β*DEXsm (2, 3-di-O-methyl-6-O-tert-butyldimethylsilyl *β*-cyclodextrin added into 14% cyanopropylphenyl /86% dimethylpolysiloxane). The oven temperature was programmed from 50°C to 220°C at 5°C/min and then held isothermal at 220°C for 5 min; injector temperature: 250°C; detector temperature: 250°C; carrier gas: H_2_ (1 mL/min); split: 1/20; injected volume: 1 *µ*L (25.9 mg of the dichloromethane extract diluted in 1 mL of chloroform).

### 3.4. NMR Spectroscopy

All NMR spectra were recorded on a Bruker AVANCE 400 Fourier-transform spectrometer operating at 400.132 MHz for ^1^H and 100.623 MHz for ^13^C, equipped with a 5 mm probe, in deuterated chloroform (CDCl_3_) with all chemical shifts referred to internal tetramethylsilane (TMS).

^13^ C NMR spectra of the hexane and dichloromethane extracts were recorded with the following parameters: flip angle 45°; acquisition time 2.66 s for the 128 K data table with a spectral width of 25000 Hz (250 ppm), relaxation delay *D*_1_ = 0.1 s (total recycling time = 2.76 s); CPD mode decoupling; digital resolution, 0.183 Hz/pt. The number of accumulated scans was 3000.

The DEPT (distortionless enhanced polarization transfer) spectrum was recorded with the same parameters, except the flip angle (135°).

^1^H NMR spectra for the quantitation of 5-hydroxymethyl-2(5H)-furanone were recorded with the following parameters: flip angle 30°; acquisition time, 2.56 s for the 32 K data table with a spectral width of 6410 Hz (16 ppm), relaxation delay *D*_1_ = 1.0 s (total recycling time = 3.56 s). Spectra were recorded with 16 scans.

Standard Bruker pulse sequences were used for 2D NMR experiments (HSQC, HMBC, and COSY).

The *T*_1_ values of the ^1^H nuclei were determined by the inversion-recovery method, using the standard sequence: 180° − *τ* − 90° − D_1_, with a relaxation delay *D*_1_ of 5.0 s. Each delay of inversion (*τ*) was thus taken into account for the computation of the corresponding *T*_1_ using the function *I*_p_ = *I*_*0*_ + *p*.e^−*τ*/T1^.

The limits of detection (LOD) and quantitation (LOQ) were determined experimentally using the signal-to-noise ratio (S : N). According to Cerceau et al. [19], the concentration for S : *N* = 10 and for S : *N* = 150 were set as LOD and LOQ, respectively. The appropriate mass of 5-hydroxymethyl-2(5H)-furanone (2.03–30.51 mg) was introduced in the NMR tube. To each of these tubes was added 0.5 mL of CDCl_3_.

## Figures and Tables

**Table 1:** Structure and NMR data of 5-hydroxymethyl-2(5H)-furanone.

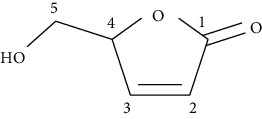					
	*δ* (^13^C ; ppm)	DEPT	*δ* (^1^H ; ppm)	HMBC	COSY
C1	173.52	C=O	—	C2 ; C3	—
C2	122.86	CH	6.16; dd	C1 ; C3 ; C4	H3
C3	153.97	CH	7.50; dd	C1 ; C2 ; C4 ; C5	H2 ; H4
C4	84.31	CH	5.17; m	C2 ; C3 ; C5	H3 ; H5
C5	62.18	CH_2_	4.01; dd 3.80; dd	C3; C4	H4

**Table 2:** Accuracy of 5-hydroxymethyl-2(5H)-furanone (F) measurements by ^1^H NMR.

Signal at 6.16 ppm (H2)				Signal at 7.50 ppm (H3)			
F area	Weighted mass (mg)	Calculated mass (mg)	RE (%)	F area	Weighted mass (mg)	Calculated mass (mg)	RE (%)
0.22	2.03	2.03	0.00	0.23	2.03	2.12	4.25
0.44	4.07	4.05	−0.49	0.44	4.07	4.05	−0.49
0.87	8.14	8.01	−1.62	0.88	8.14	8.01	−1.62
1.32	12.20	12.16	−0.33	1.32	12.20	12.16	−0.33
1.76	16.27	16.21	−0.37	1.76	16.27	16.21	−0.37
2.21	20.34	20.36	0.10	2.21	20.34	20.36	0.10
3.31	30.51	30.49	−0.07	3.31	30.51	30.49	−0.07

The area of the signal of the aldehydic proton of vanillin is fixed at 1.00 in all experiments; F area: area of the selected signals of 5-hydroxymethyl-2(5H)-furanone; mass of vanillin: 12.60 mg; calculated mass according to formula (1); RE: relative error.

**Table 3:** Precision of 5-hydroxymethyl-2(5H)-furanone (F) measurements by ^1^H NMR.

	Signal at 6.16 ppm (H2)					Signal at 7.50 ppm (H3)				
	Test 1	Test 2	Test 3	Test 4	Test 5	Test 1	Test 2	Test 3	Test 4	Test 5
Weighted mass of F (mg)	30.51	30.51	30.51	30.51	30.51	30.51	30.51	30.51	30.51	30.51
F area	3.32	3.31	3.31	3.32	3.31	3.32	3.31	3.31	3.32	3.32
Calculated mass of F (mg)	30.58	30.49	30.49	30.58	30.49	30.58	30.49	30.49	30.58	30.49
RE (%)	0.23	−0.07	−0.07	0.23	−0.07	0.23	−0.07	−0.07	0.23	−0.07

The area of the signal of the aldehydic proton of vanillin is fixed at 1.00 in all experiments; F area: area of the selected signals of 5-hydroxymethyl-2(5H)-furanone; mass of vanillin: 12.60 mg; calculated mass according to formula (1); RE: relative error.

**Table 4:** Quantitation of 5-hydroxymethyl-2(5H)-furanone (F) in the dichloromethane leaf extract from *H. lividus* subsp. *Corsicus* by ^1^H NMR.

Signal at 6.16 ppm (H2)		Signal at 7.50 ppm (H3)	
*A*_*F*_	2.84	*A*_*F*_	2.86
*m*_*F*_ (mg)	25.22	*m*_*F*_ (mg)	25.39
F content (%)	84.35	F content (%)	84.93

Mass of the extract: 29.9 mg; area of the signal of the aldehydic proton of vanillin is fixed at 1.00 in all experiments; amount of vanillin (*m*_V_) = 11.84 mg; purity of vanillin: 99%; molecular weight of 5-hydroxymethyl-2(5H)-furanone: 114.10 g·mol^−1^; molecular weight of vanillin: 152.15 g·mol^−1^; *A*_F_: areas of the selected signals at 6.16 ppm and 7.50 ppm (ethylenic protons); *m*_F_: calculated mass (mg) of F according to equation (1).

## Data Availability

No data were used to support this study.
